# Lung Function of Children at Three Sites of Varying Ambient Air Pollution Levels in Uganda: A Cross Sectional Comparative Study

**DOI:** 10.3390/ijerph15122653

**Published:** 2018-11-26

**Authors:** Bruce J. Kirenga, Rebecca Nantanda, Corina de Jong, Levicatus Mugenyi, Qingyu Meng, Gilbert Aniku, Sian Williams, Hellen Aanyu-Tukamuhebwa, Moses Kamya, Stephan Schwander, Thys van der Molen, Vahid Mohsenin

**Affiliations:** 1 Department of Medicine, Makerere University, Kampala, Uganda; mkamya@infocom.co.ug; 2 Makerere University Lung Institute, Kampala, Uganda; rnantanda@gmail.com (R.N.); lmugenyi005@gmail.com (L.M.); 3 GRIAC-Primary Care, Department of General Practice and Elderly Care, University of Groningen, University Medical Center Groningen, EB79 Groningen, The Netherlands; c.de.jong02@umcg.nl (C.d.J.); thys.v.molen@gsk.com (T.v.d.M.); 4 Departments of Urban-Global Public Health and Environmental and Occupational Health, School of Public Health, Rutgers University, The State University of New Jersey, Piscataway, NJ 08854, USA; Qingyu.Meng@dtsc.ca.gov (Q.M.); schwansk@sph.rutgers.edu (S.S.); 5 Department of Paediatrics and Child Health, Makerere University College of Health Sciences, Kampala, Uganda; gilbert.aniku@gmail.com (G.A.); hellen.aanyut@gmail.com (H.A.-T.); 6 International Primary Care Respiratory Group, Aberdeen AB32 9AE, UK; sian.health@gmail.com; 7 Department of Medicine, Yale University School of Medicine, New Haven, CT 06520, USA; vahid.mohsenin@yale.edu

**Keywords:** urbanization, lung function, air pollution, children, Uganda

## Abstract

Air pollution is a major cause of sub-optimal lung function and lung diseases in childhood and adulthood. In this study we compared the lung function (measured by spirometry) of 537 Ugandan children, mean age 11.1 years in sites with high (Kampala and Jinja) and low (Buwenge) ambient air pollution levels, based on the concentrations of particulate matter smaller than 2.5 micrometres in diameter (PM_2.5_). Factors associated with lung function were explored in a multiple linear regression model. PM_2.5_ level in Kampala, Jinja and Buwenge were 177.5 µg/m^3^, 96.3 µg/m^3^ and 31.4 µg/m^3^ respectively (*p* = 0.0000). Respectively mean forced vital capacity as % of predicted (FVC%), forced expiratory volume in one second as % of predicted (FEV_1_%) and forced expiratory flow 25–75% as % of predicted (FEF_25–75_%) of children in high ambient air pollution sites (Kampala and Jinja) vs. those in the low ambient air pollution site (Buwenge subcounty) were: FVC% (101.4%, vs. 104.0%, *p* = 0.043), FEV_1_% (93.9% vs. 98.0, *p* = 0.001) and FEF_25–75_% (87.8 vs. 94.0, *p* = 0.002). The proportions of children whose %predicted parameters were less than 80% predicted (abnormal) were higher among children living in high ambient air pollution than those living in lower low ambient air pollutions areas with the exception of FVC%; high vs. low: FEV1 < 80%, %predicted (12.0% vs. 5.3%, *p* = 0.021) and FEF_25–75_ < 80%, %predicted (37.7% vs. 29.3%, *p* = 0.052) Factors associated with lung function were (coefficient, *p*-value): FVC% urban residence (−3.87, *p* = 0.004), current cough (−2.65, *p* = 0.048), underweight (−6.62, *p* = 0.000), and overweight (11.15, *p* = 0.000); FEV_1_% underweight (−6.54, *p* = 0.000) and FEF_25–75_% urban residence (−8.67, *p* = 0.030) and exposure to biomass smoke (−7.48, *p* = 0.027). Children in study sites with high ambient air pollution had lower lung function than those in sites with low ambient air pollution. Urban residence, underweight, exposure to biomass smoke and cough were associated with lower lung function.

## 1. Introduction

Lung development commences in utero, continues through adolescence, and ceases in early adulthood (about 25 years) [1,2,3]. During this stage of growth and development, several factors such as air pollution, may affect the lungs leading to sub-optimal lung function. Sub-optimal lung function predisposes to wheezing illness as children and Chronic Obstructive Pulmonary Disease (COPD) as adults [4,5,6,7]. A study by Bui et al. that investigated the impact of childhood lung function deficits on adulthood COPD found that children in the lowest quartile of FEV_1_/FVC ratio at 7 years had almost six time the risk of COPD in middle life [6]. Prominent among the risk factors for early life lung damage is air pollution. Air pollution has the potential to impair lung growth from as early as prenatal life [8,9,10]. Children are prone to greater lung damage from air pollution than adults because of their rapidly growing and still immature lungs, more time spent outdoors and increased physical activity requiring larger inhalation volumes [11]. Studies conducted in countries other than Uganda have reported deficits in lung function and increased frequency of respiratory symptoms in children of all age groups who are exposed to air pollution (14, 16–20). Urban-rural comparisons have reported a higher frequency of respiratory symptoms and poorer lung function among the urban children [12,13,14]. One of the possible explanations is the higher degree of air pollution in the urban areas, arising from a combination of motor vehicle fumes, industries and biomass smoke arising from open rubbish burning and cooking and lighting in crowded slum areas in Low and Middle-income countries (LMICs). Like many other developing countries, Uganda is currently facing major challenges from increasing ambient and indoor air pollution levels [15,16,17]. Recently, we reported mean ambient PM_2.5_ levels in two Ugandan cities which were 5.3 times than the World Health Organization (WHO) limits [15]. Furthermore, we found that PM_2.5_ levels were higher in Kampala (the capital city) than in Jinja (the second largest city in Uganda) (138.6 μg/m^3^ vs. 99.3 μg/m^3^) [15].

We therefore hypothesized that children living in sites with high ambient air pollution (Kampala city and Jinja municipality, urban) would have lower lung function than those living the site with low ambient air pollution (Buwenge subcounty, rural). We also hypothesized that children in Jinja municipality which is a smaller city with lower ambient air pollution would have better lung function than those in Kampala city which has higher ambient air pollution. To test these hypotheses, we studied children at three sites of varying air pollution levels and urbanization in Uganda: Kampala (city), Jinja (municipality) and Buwenge subcounty (rural).

## 2. Materials and Methods

This was a cross sectional survey of school children in two urban locations: (Kampala city, Jinja municipality) and one rural site (Buwenge subcounty in Jinja district).

Kampala city is the capital city of Uganda. It is characterized by a mixture of residences, industries and commercial areas. The residents are also a mixture of high, middle and low socioeconomic status. The middle and lower class commonly use biomass for cooking, particularly in form of charcoal and wood. Jinja municipality has demographic characteristics similar to those of Kampala city but on a lower scale; the population is smaller, less vehicular traffic and fewer slums. On the hand, Buwenge subcounty, which is found in the Jinja district, is a rural area, with little vehicular traffic and residents mainly use biomass for cooking using the typical three-stone open fire stoves [18,19]. The main economic activity in Buwenge subcounty is subsistence agriculture and there are no industries.

The inhabitants of Kampala city and Jinja municipality are mainly Black Africans (99%), and the rest are either Asians or Caucasians. The people from Buwenge subcounty are all Black Africans [20]. The weather conditions in the three sites are almost the same with high temperatures and high humidity all year round. The average annual temperature is 21.9 °C and relative humidity ranges from 53–89% [21]. Annual rainfall ranges from 1750–2000 mm. The survey in Jinja municipality and Buwenge subcounty was conducted during the March–April period which is the beginning of the first rainy season (relatively little rainfall). On the other hand, we tested Kampala children during the June–July period which is the end of the first rainy season (also relatively little rain).

The study was conducted in primary schools that were randomly selected from a list obtained from the district education departments. In Kampala, the schools selected were Buganda Road primary school and Uganda Railways primary school, both in the central division of Kampala. In Jinja municipality, Victoria Nile primary school and Walukuba East primary school were selected. In Buwenge subcounty, we randomly selected Buweera and Kagoma primary schools. All the schools that were selected are day public schools. The children in public schools in both urban and rural areas are usually from the lower socioeconomic class.

### 2.1. Sample Size

The sample size calculations were based on power ≥0.80 and two-sided α = 0.05. The values used to calculate the sample size were based on estimates obtained from Asgari et al. study in Tehran [12]. We powered the study to detect a 6% difference in predicted percent FEV_1_ between urban and rural areas. Two hundred participants were needed in each of the three sites (Kampala, Jinja urban and Jinja rural) to detect this difference.

### 2.2. Recruitment

Children in in primary grades four to seven (commonly 9–12 years old) in the selected schools were all invited to participate in the study Information about the study and consent forms were sent to the parents/guardians through the children, who provided written consent to their children’s participation in the study. The children provided written assent. All participants were screened for any contraindication to spirometry (history of any mental illnesses, history of admission for cardiac illness within the last 6 months, recent thoracic, abdominal or eye surgery or retinal detachment and active tuberculosis or any other acute lung infection). Those who were found to have any contra-indications were excluded. Children with lung infections other than tuberculosis were later included upon recovery from the infection.

### 2.3. Data Collection/Procedures

A questionnaire was used to collect data on the respiratory symptoms and air pollution exposures. Children completed the questionnaire with the help of their parents or a research assistant. Data on respiratory symptoms such as wheeze, cough and breathlessness at the time of data collection and/or in the 12 months prior to enrolment were also collected. Data on indoor and outdoor air pollution exposures such as exposure to biomass smoke while cooking, burning rubbish, and second-hand tobacco smoke was also collected.

Lung function assessment: Lung function was assessed by Spirometry according to American Thoracic Society/European Respiratory Society (ATS/ERS) guidelines using a Pneumotrac^®^ with Spirotrac^®^ V software (Vitalograph Ltd., Buckingham, UK) [22]. Forced vital capacity (FVC), forced expiratory volume in the first second (FEV_1_) and forced expiratory flow at 25–75% of FVC (FEF_25–75_) and their corresponding percent predicted (%predicted) for age, weight, height, gender and ethnicity were recorded. Predicted parameters were based on NHANES III lung function prediction models [23].

Particulate pollutant measurement: PM_2.5_ concentrations were measured as an indicator of ambient air pollution. A real-time aerosol monitor, DUSTTRACK II-8530 (TSI Inc., Shoreview, MN, USA) was used to collect PM_2.5_ particle levels at each school (two schools per site). Sampling was done over 24 h for one day. 

### 2.4. Data Analysis

Descriptive statistics were used to summarize the participants’ socio-demographic characteristics and lung function parameters (forced vital capacity (FVC), forced expiratory volume in the first second (FEV_1_), FEV_1_/FVC (FEV_1_ ratio) and forced expiratory flow 25–75% (FEF_25–75_%)) and their percentage predicted values (FVC%, FEV_1_%, FEF_25–75_%). These parameters were compared between sites using analysis of variance (ANOVA) and by urban status using the independent t-test. Urban residence was defined as residing in either Kampala city or Jinja municipality while rural residence was defined as residing in Buwenge subcounty.

We analyzed for factors associated with lung function, that is, factors associated with each of percentage predicted values (FVC%, FEV_1_%, FEF_25–75_%). First, we performed simple linear regression. Factors that showed an association with the parameters with a *p*-value of ≤0.20 were all included in a multiple linear regression model. The factors assessed for association were; (1) urban status (2) Air pollution:- ambient air pollution (PM_2.5_, living within 500 m of a factory, living within 500 m of a road frequently used by cars) and exposure to biomass smoke (defined as using either charcoal, wood or kerosene in the homes for cooking or lighting), (3) tobacco smoke exposure defined as living with someone who smokes or children smoking themselves, (4) malnutrition (underweight and overweight) assessed by body mass index (BMI) for age. BMI for age was calculated using United States of America Centers for Disease Control and prevention software (children BMI tools for school) [21], (5) socioeconomic status (SES) and (6) respiratory symptoms. The SES was assessed by SES index derivation from socioeconomic parameters collected in the survey using principal component analysis (PCA) as has been previously done in other studies [24]. The variables included in the PCA analysis were occupation of parents, education level of parents, type of housing, ownership of assets and number of persons sharing a room in the household. The SES was put in 3 quintiles with the lower quintile corresponding to lower values of the index and upper quintiles corresponding to the well-off households/individuals. Age, sex and height were not included in the model for factors associated with lung function because these are corrected for in the percentage predicted parameters. Correlation between factors (multicollinearity) was checked using variance inflation factor (VIF) and centering considering in case of a multicollinearity problem (VIF > 10). A *p*-value of less than 0.05 was considered to represent significant association. All analyses were performed using Stata version 14 (StataCorp. 2015. Stata Statistical Software: Release 14, StataCorp LP, College Station, TX, USA).

### 2.5. Ethical Considerations

The study protocol was approved by the Mulago Hospital Research and Ethics Committee and the Uganda National Council for Science and Technology Administrative clearance was obtained from the departments of Education of Kampala city and Jinja district (ethical approval number: MREC:582). Parents/guardians provided informed written consent and children gave assent.

## 3. Results

### 3.1. Study Participants Characteristics

Of the 537 participants, 185 were from Kampala, 151 from Jinja Municipality and 201 from Buwenge subcounty. The survey in Jinja and Buwenge was conducted in the months of March–April 2015 (beginning of rainy season) while that in Kampala was conducted in the months of June–July 2015 (end of rainy season). Children characteristics are presented in Table 1. The mean age (standard deviation) of the children was 11.1 ± 1.3 years. The proportion of boys was 44.3% in Kampala city, 33.1% in Jinja Municipality and 54.2% in Buwenge subcounty. The mean height of urban children was significantly higher than for rural children (145.5 cm for Kampala, 144.8 cm for Jinja municipality and 139.5 cm for Buwenge subcounty, *p* = 0.0000). Urban children weighed on average 36.2 kg (36.7 kg for Kampala and 35.7 kg for Jinja municipality) compared to rural children who weighed 32.1 kg, *p* = 0.0000. Underweight rates were 10.9% for Kampala, 17.3% for Jinja and 31.5% for Buwenge. Overweight rates were 8.2% for Kampala, 2.7% for Jinja and 5.6% for Buwenge. Obesity rates were low 0.5%, 3.3% and 0.5% for Kampala, Jinja and Buwenge respectively. Having one or more of the respiratory symptoms (cough, wheeze, shortness of breath) occurred in 54.1% of the children in Kampala, 62.9% of the children in Jinja municipality and 66.7% of the children in Buwenge subcounty (*p* = 0.035).

### 3.2. Air Pollution

Air pollution exposures (indoor and outdoor) are shown in Table 2. Children had high exposures to biomass smoke in both urban and rural sites, but higher among rural children (94.6% vs. 87.1%, *p* = 0.011). Exposure to second hand tobacco smoke was higher among rural than urban children (6.2% vs. 3.0%, *p* = 0.11). The mean particulate matter pollution (PM_2.5_) levels by site are shown in Table 2. The 24-h pollution levels for Kampala city, Jinja municipality and Buwenge subcounty were 177.5 µg/m^3^, 96.3 µg/m^3^ and 31.4 µg/m^3^ respectively (*p* = 0.0000).

### 3.3. Lung Function

**Figure 1:**
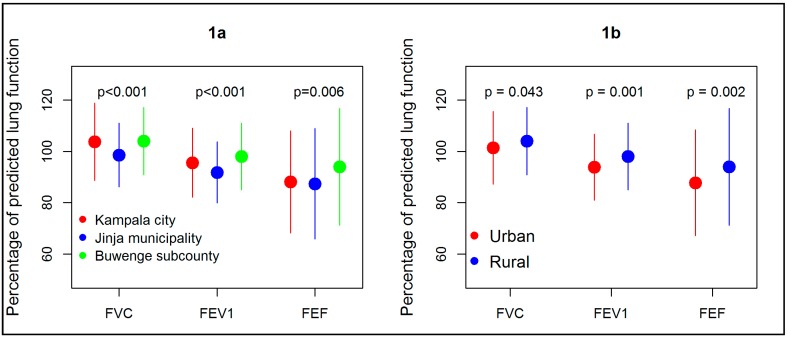
Mean distribution of spirometry parameters (percentage of predicted lung function) by site (**1a**) and (**1b**). By urban status (**1b**). Circles show mean levels and bars show standard deviation. FVC = forced vital capacity, FEVI = forced expiratory volume in one second and FEF = forced expiratory flow 25–75%.

Lung function data are presented in Table 3, Table 4 and Table 5 and Figure 1. The mean FVC, FEV_1_, FEF_25–75_% in liters for children in Kampala, Jinja and Buwenge were as follows: FVC 2.2, 2.1, 2.0, (*p* = 0.0001), FEV_1_ 2.0, 1.9, 1.8, (*p* = 0.0002) and FEF_25–75_% 2.6, 2.5, 2.5, (*p* = 0.3704) respectively. In addition, FEV_1_ ratios for Kampala, Jinja and Buwenge were 0.90, 0.91 and 0.91 respectively. The percentage predicted parameters, for Kampala, Jinja and Buwenge were: FVC% 103.7%, 98.6%, 104.0%; (*p* = 0.034), FEV_1_% 95.6%, 91.8%, 99.2% (*p* = 0.000), FEF_25–75_% 88.1%, 87.4%, 94.6% (*p* = 0.032) as highlighted in Table 3. When grouped by urban status (high vs. low air pollution levels), the actual lung function values for urban children appeared higher than for rural children but after correcting for age, height, sex (the percentage predicted parameters) rural children were found to have significantly better lung function than urban children (Table 4 and Figure 1b). A comparison the same parameters between Kampala and Jinja municipality (the two urban sites) revealed that Kampala children had higher parameters than Jinja municipality children with the exception of FEF_25–75_% where the differences where not statistically different (Table 5). The proportions of children whose %predicted parameters were less than 80% predicted by urban status urban vs. rural were: FVC < 80%, %predicted (3.3% vs. 3.0%, *p* = 0.85), FEV1 < 80%, %predicted (12.0% vs. 5.8%, *p* = 0.021) and FEF_25–75_ < 80%, %predicted (37.7% vs. 29.3%, *p* = 0.052) (Table 5).

### 3.4. Factors Associated with Lung Function 

Factors associated with each of the lung function parameter (FVC%, FEV1% and FEF_25–75_%) in a multivariate linear model are presented in Table 6. Significant associations were observed with the following factors: FVC% rural residence (3.87, *p* = 0.004), underweight (−6.62, *p* = 0.000), overweight (11.15, *p* = 0.000) and cough (−2.65, *p* = 0.048); FEV_1_% underweight (−6.54, *p* = 0.001) and FEF_25–75_% rural residence (8.67, *p* = 0.030) and exposure to biomass smoke (−7.48, *p* = 0.027).

## 4. Discussion

Results from this study show that children living in urban areas with high air pollution have significantly lower lung function and higher rates of failure to reach at least 80% of their predicted lung function than those living in less polluted rural areas. Lower lung function is associated with urban residence, underweight, cough and exposure to biomass smoke.

The finding of lower lung function in children exposed to high levels of air pollution is similar to findings from previous studies in other settings such as Greece, India and Iran [12,13,14]. A study by Asgari et al. found mean FEV_1_% of 89.0% in high ambient air pollution location and 99.0% in a low ambient air pollution areas [12]. In California a 10-year prospective study found deficits in FEV_1_ growth to be associated with pollution [25]. A follow up study in this cohort has confirmed significant improvements in lung function with decreasing air pollution (mean 4-year growth in FEV_1_ increased by 91.4 ml per decrease of 14.1 ppb in nitrogen dioxide level (*p* < 0.001), by 65.5 mL per decrease of 8.7 μg per cubic meter in PM_10_ level (*p* < 0.001), and by 65.5 mL per decrease of 12.6 μg per cubic meter in PM_2.5_ level (*p* = 0.008)) [26]. These findings suggest a deleterious effect of air pollution on children lung function [9,27,28]. Air pollution exposure is believed to result in a reduced formation of alveoli and causes inflammation that leads to airway remodeling eventually resulting in reduced lung function [29].

It must be noted that the differences in the percentage predicted parameters in our study between urban and rural children though statistically significant are much lower than observed in other studies. We found that FVC% was only 2.6% lower, FEV_1_% was 5.3% lower and FEF_25–75_% was 6.8% lower compared to the ones reported by Asgari et al. and other studies. We postulate that these smaller differences could be due to higher exposure of rural children to indoor air pollution due to more frequent use of wood for indoor cooking. Indoor air pollution exposure has also been linked to decrement in lung function. In China Roy et al. found a mean FEV_1_ of 1427 mL SD 303 mL among children exposed to biomass smoke and 1598 mL SD 325 mL among those not exposed to biomass smoke, a difference of 171 mL (10.7%) [30]. 

Although the association between lung function and air pollution (PM_2.5_) in this study did not reach statistical significance, the lower lung function in urban children in study is probably due to exposure to ambient air pollution. The average PM_2.5_ levels measured at the urban site of 141.0 µg/m^3^ far exceeds World Health Organization (WHO) defined allowable limits of 25 µg/m^3^. This is not the first study demonstrating high pollution levels in the study urban sites. In our study conducted in 2014 we demonstrated average levels of PM_2.5_ particles of 132.1 µg/m^3^ in Kampala and Jinja [15]. 

The study found that children in Jinja municipality had lower lung function than those in Kampala city which has higher ambient air pollution. We expected that lung function would be lowest in Kampala children followed by Jinja municipality and Buwenge. The reasons for this observation are not clear from the data that we have from the survey. However, we think that it probably due to the fact that Jinja municipality’s air pollution was much higher than recommended (96.3 µg/m^3^) and also had higher rates of biomass smoke exposure than Kampala city (92.7% vs. 83.6%) as well as higher rates of underweight (17.3% vs. 10.9%) and cough (51.7% vs. 46.0%).

In addition to lower attainment of expected lung function, this study found that the rates of abnormal lung function as assessed by having an FEV_1%_ and FEF_25–75_% less than 80% predicted was higher among children in high pollution urban sites. These two lung function parameters assess air narrowing or obstruction. Indeed, in children FEF_25–75_ is believed to be a better measure of airway obstruction than the FVC/FEV_1_ [31]. The abnormalities could also be reflection higher asthma which is known to be more prevalent in urban areas [32,33]. A study by Churg et al. of 20 children exposed to high air pollution in Mexico City and 20 Canadian children with low exposure to particle pollution found that Mexican children had more small airway fibrous tissue and electron microscope found high burden of particles in Mexican children with some having visible particles in the mucosa [29,34,35].

Children in urban sites, those with cough, exposure to biomass smoke and underweight had lower lung function. The association between lung function and, cough, urbanization, underweight and biomass smoke exposure has been previously reported [15,36,37,38]. In the current study, the association between biomass smoke exposure and lung function was found only for FEF_25–75_%, a key marker of small airway dysfunction. The pathophysiology of biomass smoke airway damage is not yet fully understood but a recent study in China suggests that biomass smoke probably causes disease through damage to the small airways which is consistent with the association with FEF_25–75_% observed in this study [39]. Nutritional status has been previously found to affect lung function [38,40,41,42]. In Nigeria, Kuti et al. assessed the effect of nutritional status in 250 children and found that underweight children had significantly lower lung function than normal weight children [38]. Overweight is usually associated with lower lung function. The association with overweight is probably a reflection of better wellbeing in this cohort rather than a true association because we observed high rates of underweight in this study. The association between respiratory symptoms and lower function has been previously observed and may be a reflection of obstructive diseases such as asthma [43,44]. Bremner et al. in a study in Australia showed that persons with current cough has FEV_1_ 65mls lower than those without cough [44]. 

This study did not assess physical activity which is known to affect lung function [45,46]. Jie Ji et al. in a study of 1713 Chinese girls (average age 9.9 years) found that physical activity as reported on a questionnaire was associated with better growth in mid expiratory flows per year (FEF_25–75_, 0.36 L/s vs. 0.28 L/s) (all *p* < 0.05) [45]. In another study Wang DY et al. found that lung function was positively correlated with fat free mass a surrogate of better physical activity [46]. In this study, we did not investigate physical activity and therefore cannot assess its effect on lung function. However, literature shows that rural children are usually more physically active than urban children [47,48,49].

We found that respiratory symptoms were very common among children irrespective of whether they were from high pollution (urban) or low pollution (rural). Rural children had more cough while urban children had more wheeze. The observation is consistent with that observed in other studies [12,50]. Asgari et al. found a similar pattern in Tehran: lower function among urban children and more respiratory symptoms in rural children [12]. In Bangladesh a study reports that the children in the rural area suffered significantly more from respiratory symptoms (incidence rate ratio 1.63, 95% confidence interval (CI) 1.62–1.64) [50]. The higher symptom rate among rural children could probably be due to high indoor pollution exposure. Although overall exposure to biomass used for cooking, heating and lighting in homes was high among urban and rural children, use of biomass fuels such as wood which are known to be more polluting was higher among rural children. Exposure to biomass pollution for children occurs in very high levels during cooking and probably more associated with acute respiratory effects rather than the chronic lung function deficits. Previous studies have found associations between respiratory infections and exposure to indoor air pollution. These infections could cause more symptoms especially cough and phlegm which are indicative respiratory infection rather than permanent lung function deficit [51]. Ambrose and Onyekachi analyzed Uganda demographic and health surveys of 2001 and 2011 respectively and found that acute respiratory infections were strongly associated with the use of biomass fuels in household settings [51,52]. Sanbata et al. also found that acute respiratory illness in children was correlated with living in poor housing circumstances characterized by greater biomass fuel use [52]. We further postulate that the higher reports of respiratory symptoms among the rural children in the current study could be related to poor access to health care facilities in the rural settings. Symptomatic children in urban environments may be treated earlier due to better access to healthcare hence presenting fewer concurrent symptoms. It is well described that access to healthcare is usually poor in rural areas [53,54].

Rural children were significantly shorter than urban children (145.2 cm vs. 139.5 cm). The finding that rural children are shorter has been reported in Uganda National demographic and health survey (UDHS) and the Uganda National panel surveys (UNPS) [55,56]. In the UDHS stunting rate among under five children was 35.6% among rural children and 18.6% among urban children [55] while in UNPS it was 35% vs. 15% [56]. This study did not involve a detailed assessment of factors associated with growth and nutrition. However other studies in Uganda investigating factors associated with stunting found that stunting was associated with poor health, low socioeconomic status of the family, poor education of the mother of infants < 12 months, consumption of food of low energy density (<350 kcal/100 g dry matter) and consumption of small meals [57,58].

This survey had limitations. Firstly, the survey was not conducted simultaneously at the different sites and only one day PM_2.5_ measurements were conducted. There could have been temporal changes in air pollution levels that could affect the outcomes. Children in schools were included. Children in schools may be different from their counterparts out of school in terms of socio-economic status. School going may also increase person to person transmission of respiratory infections hence the high symptom rates. Objective assessment of indoor air pollution was not done. Therefore, the true exposure status of the children to indoor pollutants cannot be ascertained. We also did not perform a detailed nutritional assessment. Nutritional is known to affect lung function and health. The study is a cross sectional survey which limits ability to study factors that could be responsible for the lower lung function.

## 5. Conclusions

Children in study sites with high ambient air pollution had lower lung function than those in sites with low ambient air pollution. Urban residence, underweight, exposure to biomass smoke and cough were associated with lower lung function.

## Figures and Tables

**Table 1:** Characteristics of study participants by site.

Characteristic	Kampala City (*n* = 185)	Jinja Municipality (*n* = 151)	Buwenge Sub-County (*n* = 201)	*p*-Value
*Socio-demographics*				
Age, Mean (SD)	11.3 (1.3)	11.0 (1.3)	11.0 (1.3)	0.2375
Gender				
Boys, (%)	44.3	33.1	54.2	0.001
Occupation of father				0.000
Professional	36.8	32.9	11.4	
Unemployed	2.7	4.1	8.3	
Peasant farmer	7.0	9.6	47.7	
Market vendor	4.9	8.2	7.3	
Builder	4.9	6.8	7.8	
Clerical worker (sales clerk, secretaries, driver etc.)	4.9	15.7	5.7	
Other	38.9	22.6	11.9	
*Anthropometry*				
Height (cm), mean (SD)	145.5 (10.0)	144.8 (8.6)	139.5 (11.6)	0.0000
Weight (kg), mean (SD)	36.7 (8.7)	35.7 (8.4)	32.1 (9.8)	0.0000
BMI categories				0.000
Underweight (BMI < 5%), %	10.9	17.3	31.5	
Normal (BMI: 5–85%), %	80.4	76.7	62.4	
Overweight (BMI: 85–95%), %	8.2	2.7	5.6	
Obese (BMI > 95%), %	0.5	3.3	0.5	
*Respiratory symptoms*				
Currently coughs several times a day, % (95% CI)	46.0 (38.7–53.2)	51.7 (43.5–59.9)	61.0 (54.2–67.9)	0.012
Wheeze% (95% CI)	23.2 (17.1–29.4)	30.7 (23.3–38.1)	21.6 (15.7–27.5)	0.131
Shortness of breath (get out of breath more easily than others), % (95% CI)	26.1 (19.7–32.5)	24.4 (17.0–31.8)	28.6 (22.0–35.2)	0.703
Has at least one respiratory symptom	54.1 (46.8–61.3)	62.9 (55.2–70.7)	66.7 (60.1–73.2)	0.035

**Table 2:** Air pollution exposures by site.

Characteristic	Kampala City (*n* = 185)	Jinja Municipality (*n* = 151)	Buwenge Sub-County (*n* = 201)	*p*-Value
***Particulate air pollutant levels in surveyed schools***				
PM_2.5_ particle levels (Minimum/Maximum)	62.5/424	30.0/236.5	16/101.5	0.0000
PM_2.5_ particle levels 24 h average, mean (SD), μg/m^3^	177.5 (43.9)	96.3(9.5)	31.4 (12.2)	0.0000
***Exposure to tobacco smoke***				
Parental current smoker (%)	4.9	0.0	4.0	0.031
Stay/live with smoker (%)	30.9	14.6	30.9	0.001
***Exposure to biomass smoke***				
Exposed to biomass used indoors (%)	83.6	92.7	94.6	0.002
Use wood for cooking/lighting (%)	16.7	51.9	97.8	0.000
Use charcoal for cooking/lighting (%)	96.1	97.9	67.9	0.000
Use kerosene for cooking/lighting (%)	23.6	30.4	36.2	0.221
Outdoor rubbish burning near home (%)	48.6	90.8	77.7	0.000
Exposed to bush burning (self/others) (%)	13.8	48.9	55.6	0.000
Lives within 500 m of industry (%)	16.1	48.9	40.7	0.000
Lives within 500 m of road used by cars (%)	89.7	79.5	73.1	0.000

**Table 3:** Lung function of study participants by site.

Characteristic	By Site			
	Kampala City (*n* = 185)	Jinja Municipality (*n* = 151)	Buwenge Sub-County (*n* = 201)	*p*-Value
**Actual lung function parameters of children unadjusted for their age, sex, and height**				
FVC (L), mean (SD)	2.2 (0.5)	2.1 (0.4)	2.0 (0.5)	0.0001
FEV_1,_ (L), mean (SD)	2.0 (0.5)	1.9 (0.5)	1.8 (0.4)	0.0002
FEV1/FVC ratio, mean (SD)	0.90 (0.09)	0.91 (0.06	0.91 (0.05)	0.000
FEF_25–75_ (mL), mean (SD)	2.6 (0.7)	2.5 (0.7)	2.5 (0.7)	0.3704
**Percentage of predicted lung function**				
FVC% predicted, mean (SD)	103.7 (15.1)	98.6 (12.4)	104.0 (13.3)	0.034
FEV1% predicted, mean (SD)	95.6 (13.5)	91.8 (11.9)	98.0 (13.0)	0.000
FEF_25–75_ predicted, mean (SD)	88.1 (19.9)	87.4 (21.5)	94.0 (22.8)	0.006
FVC < 80%, *n* (%)	5 (2.8)	6 (4.1)	6 (3.1)	0.777
FEV1 < 80%, *n* (%)	21 (11.6)	18 (12.4)	11 (5.8)	0.068
FEF_25–75_ < 80%, *n* (%)	65 (35.9)	58 (40.0)	56 (29.3)	0.113
FEV1/FVC ratio < 0.7, *n* (%)	1 (0.6)	0 (0.0)	0 (0.0)	0.629

**Table 4:** Lung function and lung function abnormalities of study participants by urban status.

Characteristic	Urban (*n* = 336)	Rural (*n* = 201)	*p*-Value
**Actual lung of children unadjusted for their age, sex, and height**			
FVC (L), mean (SD)	2.2 (0.5)	2.0 (0.5)	0.0006
FEV_1,_ (L), mean (SD)	2.0 (0.4)	1.8 (0.4)	0.001
FEV1/FVC ratio, mean (SD)	0.90 (0.05)	0.91 (0.05)	0.039
FEF_25–75_ (mL), mean (SD)	2.6 (0.7)	2.5 (0.7)	0.269
FVC% predicted, mean (SD)	101.4 (14.2)	104.0 (13.3)	0.043
FEV1% predicted, mean (SD)	93.9 (12.9)	98.0 (13.0)	0.001
FEF_25–75_ predicted, mean (SD)	87.8 (20.6)	94.0 (22.8)	0.002
FVC < 80%, *n* (%)	11 (3.4)	6 (3.1)	0.878
FEV1 < 80%, *n* (%)	39 (12.0)	11 (5.8)	0.021
FEF_25–75_ < 80%, *n* (%)	123 (37.7)	56 (29.3)	0.052
FEV1/FVC ratio < 0.7, *n* (%)	1 (0.3)	0 (0.0)	0.629

**Table 5:** Lung function of study participants comparing Kampala city and Jinja municipality.

Characteristic	Kampala City (*n* = 185)	Jinja Municipality (*n* = 151)	*p*-Value
**Actual lung of children unadjusted for their age, sex, and height**			
FVC (L), mean (SD)	2.2 (0.5)	2.1 (0.4)	0.004
FEV_1,_ (L), mean (SD)	2.0 (0.5)	1.9 (0.5)	0.011
FEV1/FVC ratio, mean (SD)	0.90 (0.09)	0.91 (0.06	0.059
FEF_25–75_ (mL), mean (SD)	2.6 (0.7)	2.5 (0.7)	0.389
FVC% predicted, mean (SD)	103.7 (15.1)	98.6 (12.4)	0.002
FEV1% predicted, mean (SD)	95.6 (13.5)	91.8 (11.9)	0.008
FEF_25–75_ predicted, mean (SD)	88.1 (19.9)	87.4 (21.5)	0.779
FVC < 80%, *n* (%)	5 (2.8)	6 (4.1)	0.494
FEV1 < 80%, *n* (%)	21 (11.6)	18 (12.4)	0.822
FEF_25–75_ < 80%, *n* (%)	65 (35.9)	58 (40.0)	0.449
FEV1/FVC ratio < 0.7, *n* (%)	1 (0.6)	0.0	0.555

**Table 6:** Factors associated with FVC%, FEV_1_% and FEF_25–75_% at multivariate stage.

Factor	Coefficient	95% CI	*p*-Value
FVC%			
Residence (ref: Urban)			
Rural	3.87	1.25–6.50	0.004
Lives within 500 m of industry	−1.28	−3.90–1.35	0.340
BMI (ref. normal):			
Under-weight	−6.62	−9.74–−3.50	0.000
Over-weight	11.15	5.55–16.75	0.000
Obese	10.44	−1.46–22.35	0.085
Current coughs several times a day	−2.65	−5.28–−0.02	0.048
Wheeze	0.09	−2.98–3.16	0.953
FEV_1_%			
Residence (ref: Urban)			
Rural	5.46	−0.10–11.02	0.054
PM_2.5_ particle levels 24 h average, μg/m^3^	−0.01	−0.05–0.02	0.491
Lives within 500 m of industry	−2.49	−5.73–0.75	0.132
BMI (ref. normal):			
Under-weight	−6.54	−10.26–−2.82	0.001
Over-weight	4.84	−1.91–11.56	0.160
Obese	9.53	−5.05–24.11	0.200
SES (ref. high):			
Low	−0.96	−6.46–4.54	0.732
Middle	−3.28	−7.19–0.63	0.100
FEF_25–75_%			
Residence (ref: Urban)			
Rural	8.67	0.8–16.49	0.030
PM_2.5_ particle levels 24 h average ^†^, μg/m^3^	0.01	−0.04–0.06	0.709
Rubbish/bush burning	4.21	−0.42–8.85	0.075
Exposure to biomass smoke	−7.48	−14.09–−0.86	0.027
Wheeze	4.52	−0.08–9.12	0.054
BMI (ref. normal):			
Under-weight	−3.71	−8.76–1.34	0.149
Over-weight	0.28	−8.71–9.28	0.951
Obese	6.55	−12.96–26.07	0.510
SES (ref. high):			
Low	1.03	−6.47–8.54	0.787
Middle	−3.33	−8.47–1.81	0.204

^†^ Centered around the mean (mean for PM_2.5_ = 100).
